# Non contiguous-finished genome sequence and description of *Enorma timonensis* sp. nov.

**DOI:** 10.4056/sigs.4878632

**Published:** 2014-02-15

**Authors:** Dhamodaran Ramasamy, Gregory Dubourg, Catherine Robert, Aurelia Caputo, Laurent Papazian, Didier Raoult, Pierre-Edouard Fournier

**Affiliations:** 1 Unité de Recherche sur les Maladies Infectieuses et Tropicales Emergentes, Institut Hospitalo-Universitaire Méditerranée-Infection, Faculté de médecine, Aix-Marseille Université; 2 Service de Réanimation Médicale, Hôpital Nord, Marseille, France; 3 Special Infectious Agents Unit, King Fahd Medical Research Center, King Abdulaziz University, Jeddah, Saudi Arabia; * Correspondence: Pierre-Edouard Fournier (pierre-edouard.fournier@univ-amu.fr)

**Keywords:** *Enorma timonensis*, genome, culturomics, taxono-genomics

## Abstract

*Enorma timonensis* strain GD5^T^ sp. nov., is the type strain of *E. timonensis* sp. nov., a new member of the genus *Enorma* within the family *Coriobacteriaceae*. This strain, whose genome is described here, was isolated from the fecal flora of a 53-year-old woman hospitalized for 3 months in an intensive care unit. *E. timonensis* is an obligate anaerobic rod. Here we describe the features of this organism, together with the complete genome sequence and annotation. The 2,365,123 bp long genome (1 chromosome but no plasmid) contains 2,060 protein-coding and 52 RNA genes, including 4 rRNA genes.

## Introduction

*Enorma timonensis* strain GD5^T^ (= CSUR P900 = DSM 26111) is the type strain of *E. timonensis* sp. nov. This bacterium was isolated from the stool of a 53-year-old French woman hospitalized for 3 months into an intensive care unit for a Guillain-Barre syndrome, as part of a culturomics study aiming at cultivating individually all species within human feces [1-3]. It is a Gram-positive, anaerobic, non-endospore forming, indole-negative, rod-shaped bacillus.

The human gut microbiota consists of billions of microorganisms that outnumber the human cells [4]. Advances in DNA sequence-based technologies and the development of 16S ribosomal RNA sequence-based metagenomic methods have been used to explore the complex gut microbial population, which has a crucial role in human health and disease development [5,6]. The currently used strategy for determining the taxonomic status of a bacterial isolate includes comparing it to its phylogenetically closest neighbors in terms of 16S rRNA gene similarity, G + C content and DNA–DNA hybridization (DDH) [7,8]. However, although considered “gold standards” in bacterial taxonomy, these criteria do not apply to all genera [9,10]. The development of high-throughput sequencing methods [11] enabled the generation of complete genomic sequences for most bacterial species of medical interest (more than 6,000 bacterial genomes sequenced to date). We recently proposed to describe new bacterial species using a polyphasic approach based on their genome sequence, MALDI-TOF spectrum and main phenotypic characteristics [12-34].

Here, we present a summary classification and a set of features for *E. timonensis* sp. nov. strain GD5^T^ (= CSUR P900 = DSM 26111) as well as the description of the complete genome sequencing and annotation. These characteristics support the circumscription of the species *E. timonensis*.

The family *Coriobacteriaceae* (Stackebrandt *et al*. 1997) was created in 1997 [35] and presently consists of 13 validated genera [36]: *Adlercreutzia* (Maruo *et al*. 2008) [37], *Asaccharobacter* (Minamida *et al*. 2008) [38], *Atopobium* (Collins and Wallbanks 1993) [39], *Collinsella* (Kageyama *et al*. 1999) [40], *Coriobacterium* (Haas and König 1988) [41], *Cryptobacterium* (Nakazawa *et al*. 1999) [42], *Denitrobacterium* (Anderson *et al*. 2000) [43], *Eggerthella* (Wade *et al*. 1999) [44], *Entherorhabdus* (Clavel *et al*. 2009) [45], *Gordonibacter* (Würdemann *et al*. 2009) [46], *Olsenella* (Dewhirst *et al*. 2001) [47], *Paraeggerthella* (Würdemann *et al*. 2009) [46], *Slackia* (Wade *et al*. 1999) [44], and the recently described new genus *Enorma* (Mishra et al. 2013) [29]. These microorganisms are anaerobic, Gram-positive, rod-shaped bacteria [42]. Members of the family *Coriobacteriaceae* are isolated from the fecal microbiota of humans or animals, and may cause infections such as bacteremia, wound infections and periodontal/endodontic infections. Members of this family also interfere with the metabolism of triglycerides, glucose, and glycogen in humans and animals [35-47].

## Classification and features

A stool sample was collected from a 53-year-old woman living in Marseille, France and hospitalized for 3 months in an intensive care unit for Guillain-Barre syndrome. She received antibiotics at the time of stool sample collection. The patient gave an informed and signed consent, and the agreement of the local ethics committee of the Institut Federatif de Recherche 48 (Marseille, France) was obtained under agreement 09-022. The fecal specimen was preserved at -80°C after collection. Strain GD5^T^ (Table 1) was isolated in 2012 by anaerobic cultivation at 37°C on 5% sheep blood-enriched Columbia agar (BioMerieux, Marcy l’Etoile, France), after 3 weeks of preincubation of the stool sample with clarified and sterile sheep rumen in an anaerobic blood culture bottle.

**Table 1:** Classification and general features of *Enorma timonensis* strain GD5^T^ according to the MIGS recommendations [48]

**MIGS ID**	**Property**	**Term**	**Evidence code^a^**
		Domain *Bacteria*	TAS [49]
		Phylum *Actinobacteria*	TAS [50,51]
		Class *Actinobacteria*	TAS [35]
	Current classification	Order *Coriobacteriales*	TAS [29,52,53]
		Family *Coriobacteriaceae*	TAS [52,53]
		Genus *Enorma*	TAS [53]
		Species *Enorma timonensis*	IDA
		Type strain GD5^T^	IDA
	Gram stain	positive	IDA
	Cell shape	rod	IDA
	Motility	non motile	IDA
	Sporulation	non sporulating	IDA
	Temperature range	mesophile	IDA
	Optimum temperature	37°C	IDA
MIGS-6.3	Salinity	unknown	IDA
MIGS-22	Oxygen requirement	anaerobic	IDA
	Carbon source	unknown	NAS
	Energy source	unknown	NAS
MIGS-6	Habitat	human gut	IDA
MIGS-15	Biotic relationship	free living	IDA
MIGS-14	Pathogenicity Biosafety level Isolation	Unknown 2 human feces	
MIGS-4	Geographic location	France	IDA
MIGS-5	Sample collection time	January 2012	IDA
MIGS-4.1	Latitude	43.296482	IDA
MIGS-4.1	Longitude	5.36978	IDA
MIGS-4.3	Depth	Surface	IDA
MIGS-4.4	Altitude	0 m above sea level	IDA

Evidence codes – IDA: Inferred from Direct Assay; TAS: Traceable Author Statement (i.e., a direct report exists in the literature); NAS: Non-traceable Author Statement (i.e., not directly observed for the living, isolated sample, but based on a generally accepted property for the species, or anecdotal evidence). These evidence codes are from the Gene Ontology project [54]. If the evidence is IDA, then the property was directly observed for a live isolate by one of the authors or an expert mentioned in the acknowledgements.

**Figure 1:**
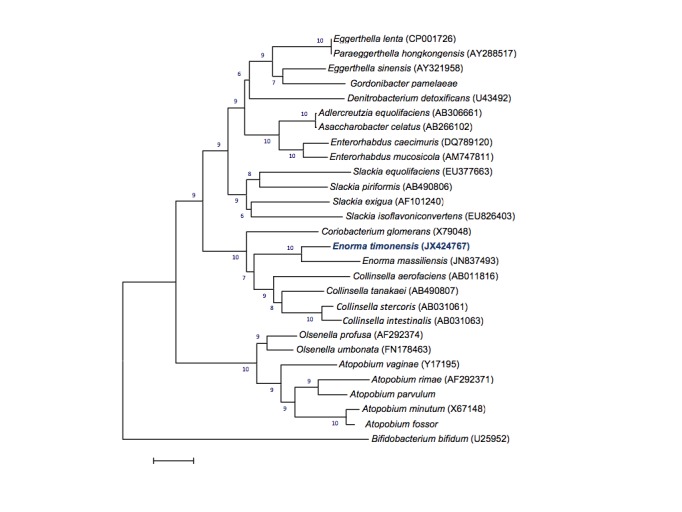
Phylogenetic tree highlighting the position of *Enorma timonensis* strain GD5^T^ relative to other type strains within the *Coriobacteriaceae* family. GenBank accession numbers are indicated in parentheses. Sequences were aligned using CLUSTALW, and phylogenetic inferences obtained using the maximum-likelihood method within the MEGA software. Numbers at the nodes are percentages of 500 bootstrap replicates supporting that node. The tree is a majority consensus tree. *Bifidobacterium bifidum* was used as outgroup. The scale bar represents a 2% nucleotide sequence divergence.

The 16S rDNA sequence (GenBank accession number JX424767) of *E. timonensis* strain GD5^T^ exhibited the highest similarity (95.0%) with its phylogenetically closest published species, *Enorma massiliensis* (Figure 1). By comparison with the type species of genera from the family *Coriobacteriaceae*, *E. timonensis* exhibited a 16S rDNA sequence similarity ranging from 84 to 95%. This value was lower than the 98.7% 16S rDNA gene sequence threshold recommended by Stackebrandt and Ebers to delineate a new species without carrying out DNA-DNA hybridization [8].

**Figure 2:**
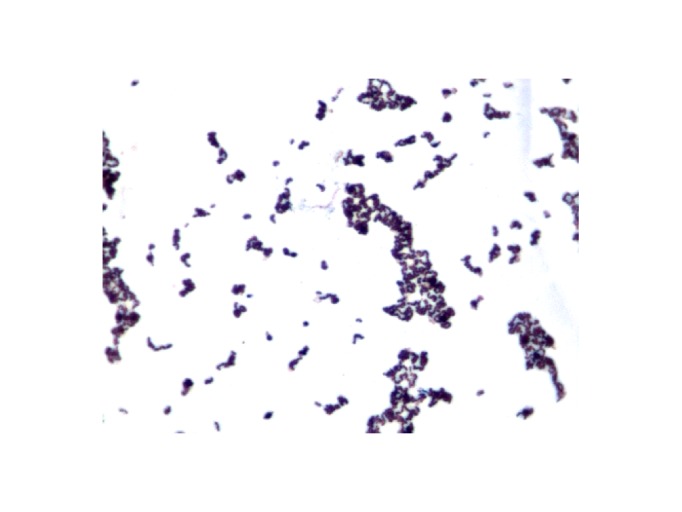
Gram staining of *E. timonensis* strain GD5^T^.

**Figure 3:**
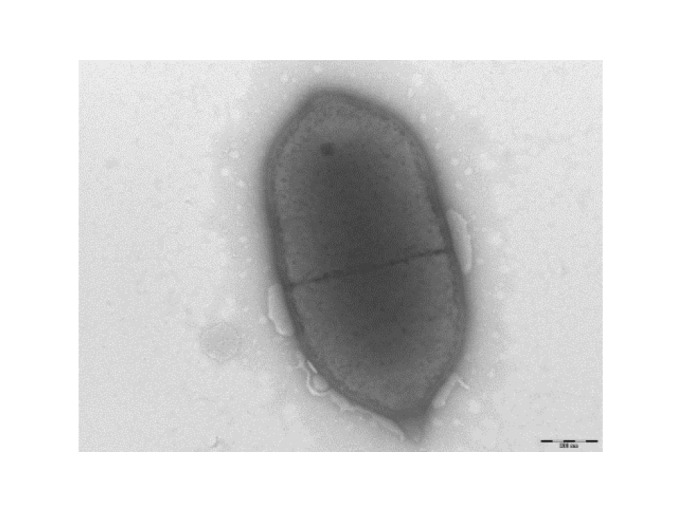
Transmission electron microscopy of *E. timonensis* strain GD5^T^ using a Morgani 268D (Philips) at an operating voltage of 60kV. The scale bar represents 200µm.

Growth at different temperatures (25, 30, 37, 45°C) was tested. No growth was observed at 25°C or 30°C. Growth occurred at both 37 and 45°C, but optimal growth was observed at 37°C after 48 hours of incubation. Colonies were translucent grey and approximately 0.4 mm in diameter on 5% sheep blood-enriched Columbia agar (BioMerieux). Growth of the strain was tested in blood-enriched Columbia agar under anaerobic and microaerophilic conditions using GENbag anaer and GENbag microaer systems, respectively (BioMerieux), and under aerobic conditions, with or without 5% CO2. Growth was achieved only anaerobically. Gram staining showed Gram-positive and non-sporulated rods (Figure 2). A motility test was negative. Cells grown on agar have a mean diameter of 0.58 µm and a mean length of 1.32µm, and are mostly grouped in short chains or small clumps (Figure 3).

Strain GD5^T^ exhibited neither catalase nor oxidase activities (Table 2). Using an API ZYM strip (BioMerieux), positive reactions were observed for leucine arylamidase, valine arylamidase, cystin arylamidase, naphthol-AS-BI-phosphohydrolase, β-galactosidase, β-glucuronidase, α-glucosidase and β-glucosidase. Negative reactions were observed for acid phosphatase, nitrate reduction, urease alkaline phosphatase, esterase (C4), esterase lipase (C8), lipase (C14), trypsin, α-chemotrypsin, acid phosphatase, α-galactosidase, N-actetyl-β-glucosaminidase, α-mannosidase, α-fucosidase. Using an API Rapid ID 32A strip (BioMerieux), positive reactions were observed for proline arylamidase, phenylalanine arylamidase, histidin arylamidase, serine arylamidase. Negative reactions were observed for urease, arginine dihydrolase, tyrosin arylamidase, leucyl-glycyl arylamidase, alanine arylamidase, glycine arylamidase and arginine arylamidase. Using an API 50 CH strip (BioMerieux), negative reactions were recorded for fermentation of glycerol, erythritol, D-arabinose, L-arabinose, D-ribose, D-xylose, L-xylose, D-adonitol, methyl-βD-xylopranoside, D-galactose, D-glucose, D-fructose, D-mannose, L-sorbose, L-rhamnose, dulcitol, inositol, D-mannitol, D-sorbitol, methyl-α-D-xylopyranoside, methyl-α-D-glucopyranoside, N-acetylglucosamine, amygdalin, arbutin, esculin ferric citrate, salicin, D-cellobiose, D-maltose, D-lactose, D-mellibiose, D-saccharose, D-trehalose, inulin, D-melezitose, D-raffinose, amidon, glycogen, xylitol, gentiobiose, D-turanose, D-lyxose, D-tagatose, D-fucose, L-fucose, D-arabitol, L-arabitol, potassium gluconate, potassium 2-ketogluconate, potassium-5-ketogluconate.

**Table 2:** Differential characteristics of *Enorma timonensis* GD5^T^, *Enorma massiliensis* strain phI^T^, *Collinsella aerofaciens* strain YIT 10235^T^, *Collinsella tanakei* strain YIT 12064^T^ and *Coriobacterium glomerans* strain PW2.

Properties	*E. timonensis*	*E. massiliensis*	*C. aerofaciens*	*C. tanakei*	*C. glomerans*
Cell diameter (µm)	0.58	0.57	0.3 – 0.7	0.5	NA
Oxygen requirement	anaerobic	anaerobic	anaerobic	anaerobic	anaerobic
Gram stain	+	+	+	+	+
Salt requirement	na	na	na	na	na
Motility	-	-	na	-	-
Endospore formation	-	-	-	na	-
Production of					
Alkaline phosphatase	-	-	-	+	na
Acid phosphatase	-	na	-	+	na
Catalase	-	-	na	-	na
Oxidase	-	-	na	-	na
Nitrate reductase	-	-	na	-	na
Urease	-	-	-	-	na
α-galactosidase	+	+	-	-	na
β-galactosidase	+	+	+	-	na
β-glucuronidase	-	-	-	+	na
α -glucosidase	+	+	+	-	na
β-glucosidase	+	+	-	+	na
Esterase	-	na	-	-	na
Esterase lipase	-	na	-	-	na
Indole	-	-	na	-	na
N-acetyl-β-glucosaminidase	-	-	-	-	na
6-Phospho-β -galactosidase	-	-	-	-	na
Arginine arylamidase	+	+	+	+	na
glutamic acid decarboxylase	-	-	-	-	na
Leucyl glycine arylamidase	-	-	+	+	na
Alanine arylamidase	-	-	-	-	na
Proline arylamidase	+	+	+	+	na
Serine arylamidase	+	-	-	-	na
Tyrosine arylamidase	-	-	-	-	na
Glycine arylamidase	-	-	+	+	na
Utilization of					
D-mannose	-	+	+	+	+
Habitat	human gut	human gut	human gut	human gut	na

na: data not available

+/-: depending on tests used

*E. timonensis* is susceptible to amoxicillin-clavulanic acid, metronidazole, imipenem, vancomycin, rifampicin, gentamicin and resistant to penicillin G, amoxicillin, ceftriaxon, erythromycin, and trimethoprim/sulfamethoxazole.

By comparison with *E. massiliensis*, *E. timonensis* differed in production of serine arylamidase and mannose fermentation, as well as susceptibility to amoxicillin.

**Figure 4:**
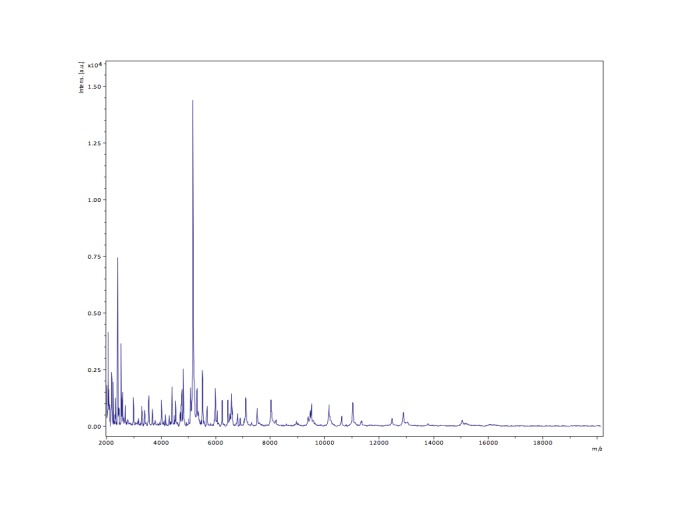
Reference mass spectrum from *E. timonensis* strain GD5^T^. Spectra from 12 individual colonies were compared and a reference spectrum was generated.

**Figure 5:**
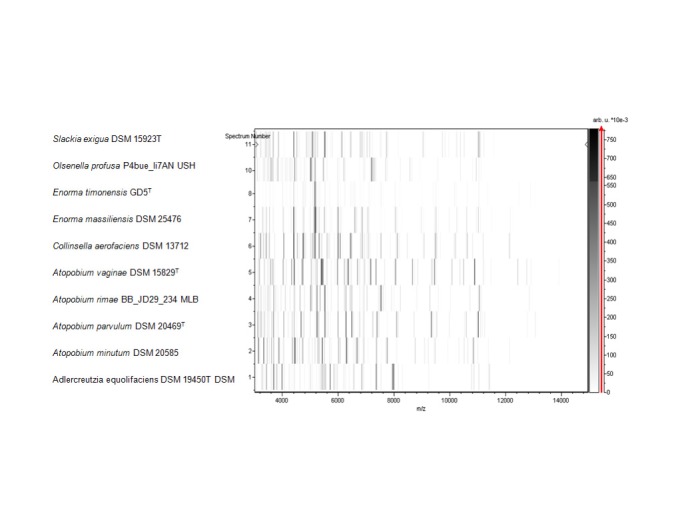
Gel view comparing *E. timonensis* sp. nov strain GD5^T^ and other members of the *Coriobacteriaceae* family. The gel view displays the raw spectra of all loaded spectrum files arranged in a pseudo-gel like look. The x-axis records the m/z value. The left y-axis displays the running spectrum number originating from subsequent spectra loading. The peak intensity is expressed by a gray scale scheme code. The color bar and the right y-axis indicate the relation between the color a peak is displayed with and the peak intensity in arbitrary units. Displayed species are indicated on the left.

Matrix-assisted laser-desorption/ionization time-of-flight (MALDI-TOF) MS protein analysis was carried out as previously described [55] using a Microflex spectrometer (Bruker Daltonics, Leipzig, Germany). Twelve distinct deposits were done for strain GD5^T^ from twelve isolated colonies. The twelve GD5^T^ spectra were imported into the MALDI BioTyper software (version 2.0, Bruker) and analyzed by standard pattern matching (with default parameter settings) against the main spectra of 4,706 bacteria, which were used as reference data, in the BioTyper database. For strain GD5^T^, no significant score was obtained, thus suggesting that our isolate was not a member of a known species. We added the spectrum from strain GD5^T^ to our database (Figure 4, Figure 5).

## Genome sequencing information

### Genome project history

The organism was selected for sequencing on the basis of its phylogenetic position and 16S rDNA similarity to *E. massiliensis* and other members of the family *Coriobacteriaceae* and is part of a study of the human digestive flora aiming at isolating all bacterial species within human feces [1-3]. It was the 2^nd^ genome of an *Enorma* species and the first genome of *E. timonensis* sp. nov. The GenBank accession number is CAPF00000000 and consists of 105 contigs. Table 3 shows the project information and its association with MIGS version 2.0 compliance [48].

**Table 3:** Project information

**MIGS ID**	**Property**	**Term**
MIGS-31	Finishing quality	High-quality draft
MIGS-28	Libraries used	One paired-end 454 3-kb library
MIGS-29	Sequencing platforms	454 GS FLX Titanium
MIGS-31.2	Fold coverage	43.5
MIGS-30	Assemblers	Newbler version 2.5.3
MIGS-32	Gene calling method	Prodigal
	INSDC ID	PRJEB543
	GenBank ID	CAPF00000000
	GenBank Date of Release	April 25, 2013
MIGS-13	Project relevance	Study of the human gut microbiome

### Growth conditions and DNA isolation

*Enorma timonesis* sp. nov., strain GD5^T^ (= CSUR P900 = DSM 26111), was grown anaerobically on 5% sheep blood-enriched Columbia agar (BioMerieux) at 37°C. Four Petri dishes were spread and resuspended in 1ml TE buffer prior to being treated with 2.5 µg/µL lysozyme for 30 minutes at 37°C, and then with Proteinase K overnight at 37°C. The DNA was then purified by 3 successive phenol-chloroform extractions followed by an ethanol precipitation at -20°C overnight. Following centrifugation, the DNA was then resuspended in 305 µL TE buffer. The DNA was then concentrated and purified using a QIAamp kit (Qiagen). The yield and concentration was measured by the Quant-it Picogreen kit (Invitrogen) on the Genios Tecan fluorometer at 66.5 ng/µl.

### Genome sequencing and assembly

DNA (5 µg) was mechanically fragmented on a Hydroshear device (Digilab, Holliston, MA, USA) with an enrichment size at 3-4kb. The DNA fragmentation was visualized through the Agilent 2100 BioAnalyzer on a DNA labchip 7500 with an optimal size of 4.4kb. A 3kb paired-end library was constructed according to the 454 GS FLX Titanium paired-end protocol (Roche). Circularization and nebulization were performed and generated a pattern with an optimal at 470 bp. After PCR amplification through 17 cycles followed by double size selection, the single stranded paired-end library was then quantified on the Agilent 2100 BioAnalyzer on a RNA pico 6000 LabChip at 136 pg/µL. The library concentration equivalence was calculated as 5.31E+08 molecules/µL. The library was stored at -20°C until further use.

The paired-end library was clonally amplified with 0.5cpb and 2cbp in 2 SV-emPCR with the GS Titanium SV-emPCR Kit (Lib-L) v2 (Roche). The yields of the emPCRs were 9.37 and 14.09%, respectively, in the range of 5 to 20% from the Roche procedure.

Approximately 790,000 beads were loaded on 1/4 region of a GS Titanium PicoTiterPlate PTP Kit 70x75 and sequenced with the GS-FLX Titanium Sequencing Kit XLR70 (Roche). The run was performed overnight and then analyzed on the cluster through the gsRunBrowser and gsAssembler (Roche). A total of 282,633 passed filter wells were obtained and generated 102.68Mb with a length average of 363 bp. The globally passed filter sequences were assembled using Newbler with 90% identity and 40bp as overlap. The final assembly identified 5 scaffolds and 105 large contigs (>1,500 bp) generating a genome size of 2.36 Mb which corresponds to a coverage of 43.5 genome equivalents.

### Genome annotation

Open Reading Frames (ORFs) were predicted using Prodigal [56] with default parameters. However, the predicted ORFs were excluded if they spanned a sequencing gap region. The predicted bacterial protein sequences were searched against the GenBank [57] and Clusters of Orthologous Groups (COG) databases using BLASTP. The tRNAs and rRNAs were predicted using the tRNAScanSE [58] and RNAmmer [59] tools, respectively. Lipoprotein signal peptides and numbers of transmembrane helices were predicted using SignalP [60] and TMHMM [61], respectively. ORFans were identified if their BLASTP *E*-value was lower than 1e-03 for alignment length greater than 80 amino acids. If alignment lengths were smaller than 80 amino acids, we used an *E*-value of 1e-05. Such parameter thresholds have already been used in previous works to define ORFans. Artemis [62] and DNA Plotter [63] were used for data management and visualization of genomic features, respectively. The Mauve alignment tool (version 2.3.1) was used for multiple genomic sequence alignment [64]. To estimate the mean level of nucleotide sequence similarity at the genome level between *E. timonensis* and five other members of the family *Coriobacteriaceae* (Table 6), we used the Average Genomic Identity Of gene Sequences (AGIOS) home-made software. Briefly, this software combines the Proteinortho software [65] for detecting orthologous proteins between genomes compared two by two, then retrieves the corresponding genes and determines the mean percentage of nucleotide sequence identity among orthologous ORFs using the Needleman-Wunsch global alignment algorithm. *Enorma timonensis* strain GD5^T^ was compared to *E. massiliensi* strain phI^T^ (GenBank accession number CAGZ00000000), *C. aerofaciens* strain ATCC 25986 (AAVN00000000), *C. tanakei* strain YIT 12063 (ADLS00000000) and *C. glomerans* strain PW2 (NC_015389).

## Genome properties

**Figure 6:**
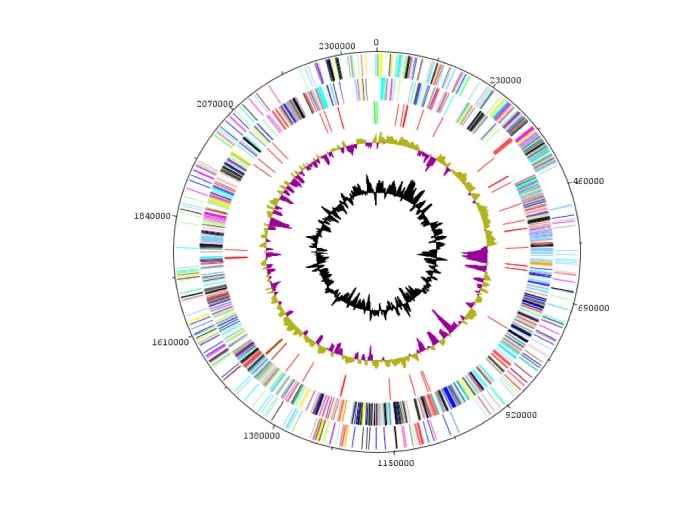
Graphical circular map of the chromosome. From the outside in: genes on the forward strand (colored by COG categories), genes on the reverse strand (colored by COG categories), RNA genes (rRNAs green, tRNAs red), GC skew (purple: negative values, olive: positive values), and G+C content plot.

The genome is 2,365,123 bp long (1 chromosome, no plasmid) with a 65.8% G+C content (Figure 6 and Table 4). Of the 2,060 predicted chromosomal genes, 2,006 were protein-coding genes and 52 were RNAs, including a complete rRNA operon, an additional 5S rRNA and 48 tRNAs. A total of 1,384 genes (67.18%) were assigned a putative function. Fifty-five genes were identified as ORFans (2.74%) and the remaining genes were annotated as hypothetical proteins. The properties and statistics of the genome are summarized in Tables 3 and 4. The distribution of genes into COGs functional categories is presented in Table 5.

**Table 4:** Nucleotide content and gene count levels of the genome

**Attribute**	Value	% of total^a^
Genome size (bp)	2,365,123	
DNA coding region (bp)	2,061,753	87.17
DNA G+C content (bp)	1,556,250	65.8
Total genes	2,060	100
RNA genes	52	2.62
Protein-coding genes	2,006	97.37
Genes with function prediction	1,384	67.18
Genes assigned to COGs	1,518	73.68
Genes with peptide signals	88	4.27
Genes with transmembrane helices	466	22.62

^a^ The total is based on either the size of the genome in base pairs or the total number of protein coding genes in the annotated genome

**Table 5:** Number of genes associated with the 25 general COG functional categories

**Code**	**Value**	**% of total**^a^	**Description**
J	140	6.98	Translation
A	0	0	RNA processing and modification
K	152	7.58	Transcription
L	90	4.48	Replication, recombination and repair
B	1	0.05	Chromatin structure and dynamics
D	20	1.0	Cell cycle control, mitosis and meiosis
Y	0	0	Nuclear structure
V	52	2.59	Defense mechanisms
T	61	3.04	Signal transduction mechanisms
M	92	4.58	Cell wall/membrane biogenesis
N	3	0.15	Cell motility
Z	0	0	Cytoskeleton
W	0	0	Extracellular structures
U	16	0.80	Intracellular trafficking and secretion
O	45	2.24	Posttranslational modification, protein turnover, chaperones
C	78	3.88	Energy production and conversion
G	224	11.16	Carbohydrate transport and metabolism
E	172	8.57	Amino acid transport and metabolism
F	50	2.49	Nucleotide transport and metabolism
H	40	1.99	Coenzyme transport and metabolism
I	38	1.89	Lipid transport and metabolism
P	71	3.54	Inorganic ion transport and metabolism
Q	13	0.65	Secondary metabolites biosynthesis, transport and catabolism
R	205	10.22	General function prediction only
S	118	5.88	Function unknown
-	488	24.32	Not in COGs

^a^ The total is based on the total number of protein coding genes in the annotated genome

## Genome comparison of *E. timonensis* with other members of the *Coriobacteriaceae* family

We compared the genome of *E. timonensis* strain GD5^T^ with those of *E. massiliensis* phI, *Collinsella aerofaciens* strain ATCC 25986, *Collinsella tanakaei* strain YIT 12063 and *Coriobacterium glomerans* strain PW2 (Table 6).

**Figure 7:**
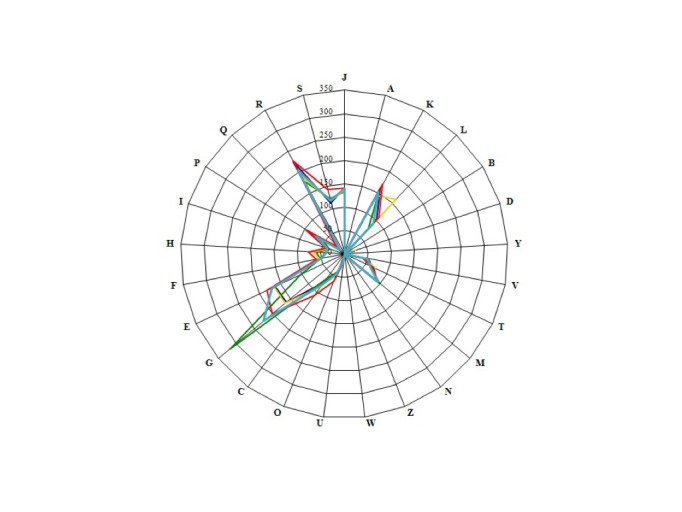
Distribution of functional classes of predicted genes in the *E. timonensis* (colored in light blue), *E. massiliensis* (dark blue), *Coriobacterium glomerans* (green), *Colinsella aerofaciens* (yellow) and *Colinsella tanakaei* (red) chromosomes, according to the clusters of orthologous groups of proteins.

The draft genome sequence of *E. timonensis* strain GD5^T^ is smaller than those of *C. aerofaciens and C. tanakaei* (2.36, 2.43 and 2.48 Mb, respectively), but larger than those of *E. massiliensis* and *C. glomerans* (2.26 and 2.11 Mb, respectively). The G+C content of *E. timonensis* is larger than those of *E. massiliensis*, *C. aerofaciens*, *C. tanakaei* and *C. glomerans* (65.80, 62.0, 60.54, 60.23 and 60.40%, respectively). The gene content of *E. timonensis* is smaller to those of *E. massiliensis*, *C. glomerans* and *C. tanakaei* (2,006, 2,159 and 2,195, respectively) but larger than those of *C. aerofaciens* and *C. tanakaei* (1,901 and 1,768, respectively). The distribution of genes into COG categories was not entirely similar in all compared genomes (Figure 7).

In addition, *E. timonensis* shared 1,109, 1,026, 880 and 1,077 orthologous genes with *E. massiliensis, C. aerofaciens, C. glomerans and C. tanakaei* respectively. The average genomic nucleotide sequence identity ranged from 66.37 to 79.44% among *Coriobacteriaceae* family members, and from 66.01 to 79.44% between *E. timonensis* and other species (Table 6A and Table 6B).

**Table 6A:** Genomic comparison of *E. timonensis* and four other members of the *Coriobacteriaceae family*.^†^

**Species**	**Strain**	**Genome accession number**	**Genome size (Mb)**	**G+C content**
*E. timonensis*	GD5^T^	CAPF00000000	2,365,123	65.80
*E. massiliensis*	phI	CAGZ01000000	2,263,008	62.0
*C. aerofaciens*	ATCC 25986	AAVN00000000	2,439,869	60.54
*C. glomerans*	PW2	NC_015389	2,115,681	60.40
*C. tanakaei*	YIT 12063	ADLS00000000	2,482,197	60.23

^†^Species and strain names, Genome GenBank accession numbers, Genome sizes and G+C contents of compared genomes.

**Table 6B:** Genomic comparison of E. timonensis and four other members of the Coriobacteriaceae family.^†^

	*E. timonensis*	*E. massiliensis*	*C. aerofaciens*	*C. glomerans*	*C. tanakaei*
*E. timonensis*	**2,006**	1109	1026	880	1077
*E. massiliensis*	79.44	**1,901**	1046	899	1103
*C. aerofaciens*	66.37	66.01	**2,159**	880	1062
*C. glomerans*	73.39	72.38	66.15	**1,768**	913
*C. tanakaei*	74.02	73.43	64.96	71.27	**2,195**

**†**Numbers of orthologous protein shared between genomes (upper right), average percentage similarity of nucleotides corresponding to orthologous protein shared between genomes (lower left) and numbers of proteins per genome (bold).

## Conclusion

On the basis of phenotypic, phylogenetic and genomic analyses (taxono-genomics), we formally propose the creation of *Enorma timonensis* sp. nov. that contains strain GD5^T^. This bacterium has been found in France.

## Description of *Enorma timonensis* sp. nov.

*Enorma timonensis* (ti.mo.nen’sis. L. gen. masc. *timonensis*, of Timone, the name of the hospital where strain GD5^T^ was cultivated). Colonies are translucent grey and 0.4 mm in diameter on blood-enriched Columbia agar. Cells are rod-shaped with a mean diameter of 0.58 µm and a mean length of 1.32 µm. Optimal growth is achieved in anaerobic conditions. No growth is observed in aerobic or microaerophilic conditions. Growth occurs between 37-45°C, with optimal growth being observed at 37°C on blood-enriched Columbia agar. Cells are Gram-positive, non-endospore forming, and non-motile. Cells are negative for catalase and oxidase. Using an API ZYM strip, positive reactions are observed for leucine arylamidase, valine arylamidase, cystin arylamidase, naphthol-AS-BI-phosphohydrolase, β-galactosidase, β-glucuronidase, α-glucosidase and β-glucosidase. Negative reactions are observed for acid phosphatase, nitrate reduction, urease alkaline phosphatase, esterase (C4), esterase lipase (C8), lipase (C14), trypsin, α-chemotrypsin, acid phosphatase, α-galactosidase, N-actetyl-β-glucosaminidase, α-mannosidase, α-fucosidase. Using an API Rapid ID 32A strip, positive reactions are observed for proline arylamidase, phenylalanine arylamidase, histidin arylamidase, serine arylamidase. Negative reactions are observed for urease, arginine dihydrolase, tyrosin arylamidase, leucyl-glycyl arylamidase, alanine arylamidase, glycine arylamidase and arginine arylamidase. Using an API 50 CH strip, fermentation or assimilation was not observed.

Cells are susceptible to amoxicillin-clavulanic acid, metronidazole, imipenem, vancomycin, rifampicin, gentamicin and resistant to penicillin G, amoxicillin, ceftriaxon, erythromycin, and trimethoprim/sulfamethoxazole. The 16S rDNA and genome sequences are deposited in GenBank under accession numbers JX424767 and CAPF00000000, respectively. The G+C content of the genome is 65.8%. The habitat of the organism is the human digestive tract. The type strain GD5^T^ (= CSUR P900 = DSM 26111) was isolated from the fecal flora of a 53-year old French patient hospitalized in an intensive care unit. This strain has been found in Marseille, France.

## References

[1] Lagier JC Armougom F Million M Hugon P Pagnier I Robert C Bittar F Fournous G Gimenez G Maraninchi M Microbial culturomics: paradigm shift in the human gut microbiome study. Clin Microbiol Infect 2012; 18:1185-1193 23033984 10.1111/1469-0691.12023

[2] Dubourg G Lagier JC Armougom F Robert C Hamad I Brouqui P The gut microbiota of a patient with resistant tuberculosis is more comprehensively studied by culturomics than by metagenomics. Eur J Clin Microbiol Infect Dis 2013; 32:637-645 10.1007/s10096-012-1787-3 23291779

[3] Pfleiderer A, Lagier JC, Armougom F, Robert C, Vialettes B, Raoult D. Culturomics identified 11 new bacterial species from a single anorexia nervosa stool sample. [Epub ahead of print]. *Eur J Clin Microbiol Infect Dis* 2013. 10.1007/s10096-013-1900-2 23728738

[4] Whitman WB Coleman DC Wiebe WJ Prokaryotes: the unseen majority. Proc Natl Acad Sci USA 1998; 95:6578-6583 10.1073/pnas.95.12.6578 9618454 PMC33863

[5] Qin J Li R Raes J Arumugam M Burgdorf KS Manichanh C Nielsen T Pons N Levenez F Yamada T A human gut microbial gene catalogue established by metagenomic sequencing. Nature 2010; 464:59-65 10.1038/nature08821 20203603 PMC3779803

[6] Clemente JC Ursell LK Parfrey LW Knight R The impact of the gut microbiota on human health: an integrative view. Cell 2012; 148:1258-1270 10.1016/j.cell.2012.01.035 22424233 PMC5050011

[7] Tindall BJ Rossello-Mora R Busse HJ Ludwig W Kampfer P Notes on the characterization of prokaryote strains for taxonomic purposes. Int J Syst Evol Microbiol 2010; 60:249-266 10.1099/ijs.0.016949-0 19700448

[8] Stackebrandt E Ebers J Taxonomic parameters revisited: tarnished gold standards. Microbiol Today 2006; 33:152-155

[9] Wayne LG Brenner DJ Colwell RR Grimont PAD Kandler O Krichevsky MI Moore LH Moore WEC Murray RGE Stackebrandt E Report of the ad hoc committee on reconciliation of approaches to bacterial systematic. Int J Syst Bacteriol 1987; 37:463-464 10.1099/00207713-37-4-463

[10] Rossello-Mora R. DNA-DNA Reassociation Methods Applied to Microbial Taxonomy and Their Critical Evaluation. In: Stackebrandt E (ed), Molecular Identification, Systematics, and population Structure of Prokaryotes. Springer, Berlin, 2006; p. 23-50.

[11] Welker M Moore ER Applications of whole-cell matrix-assisted laser-desorption/ionization time-of-flight mass spectrometry in systematic microbiology. Syst Appl Microbiol 2011; 34:2-11 10.1016/j.syapm.2010.11.013 21288677

[12] Kokcha S Mishra AK Lagier JC Million M Leroy Q Raoult D Fournier PE Non-contiguous finished genome sequence and description of *Bacillus timonensis* sp. nov. Stand Genomic Sci 2012; 6:346-355 10.4056/sigs.2776064 23408487 PMC3558959

[13] Lagier JC El Karkouri K Nguyen TT Armougom F Raoult D Fournier PE Non-contiguous finished genome sequence and description of *Anaerococcus senegalensis* sp. nov. Stand Genomic Sci 2012; 6:116-125 10.4056/sigs.2415480 22675604 PMC3359877

[14] Mishra AK Gimenez G Lagier JC Robert C Raoult D Fournier PE Non-contiguous finished genome sequence and description of *Alistipes senegalensis* sp. nov. Stand Genomic Sci 2012; 6:304-314 10.4056/sigs.2625821 PMC3558963 23407265

[15] Lagier JC Armougom F Mishra AK Ngyuen TT Raoult D Fournier PE Non-contiguous finished genome sequence and description of *Alistipes timonensis* sp. nov. Stand Genomic Sci 2012; 6:315-324. http//dx..org/10.4056/sigs.2685971 23408657 10.4056/sigs.2685971 PMC3558960

[16] Mishra AK Lagier JC Robert C Raoult D Fournier PE Non-contiguous finished genome sequence and description of *Clostridium senegalense* sp. nov. Stand Genomic Sci 2012; 6:386-395. http//dx..org/ 10.4056/sigs.2766062 23408737 10.4056/sigs.2766062 PMC3558962

[17] Mishra AK Lagier JC Robert C Raoult D Fournier PE Non-contiguous finished genome sequence and description of *Peptoniphilus timonensis* sp. nov. Stand Genomic Sci 2012; 7:1-11 10.4056/sigs.2956294 23449949 PMC3570796

[18] Mishra AK Lagier JC Rivet R Raoult D Fournier PE Non-contiguous finished genome sequence and description of *Paenibacillus senegalensis* sp. nov. Stand Genomic Sci 2012; 7:70-81 10.4056/sigs.305645023459006 10.4056/sigs.3056450 PMC3577113

[19] Lagier JC Gimenez G Robert C Raoult D Fournier PE Non-contiguous finished genome sequence and description of *Herbaspirillum massiliense* sp. nov. Stand Genomic Sci 2012; 7:200-20910.4056/sigs.308647423407294 10.4056/sigs.3086474 PMC3569391

[20] Kokcha S Ramasamy D Lagier JC Robert C Raoult D Fournier PE Non-contiguous finished genome sequence and description of *Brevibacterium senegalense* sp. nov. Stand Genomic Sci 2012; 7:233-245 10.4056/sigs.3256677 23408786 PMC3569389

[21] Ramasamy D Kokcha S Lagier JC N’Guyen TT Raoult D Fournier PE Non-contiguous finished genome sequence and description of *Aeromicrobium massilense* sp. nov. Stand Genomic Sci 2012; 7:246-257 10.4056/sigs.3306717 23408663 PMC3569385

[22] Lagier JC Ramasamy D Rivet R Raoult D Fournier PE Non-contiguous finished genome sequence and description of *Cellulomonas massiliensis* sp. nov. Stand Genomic Sci 2012; 7:258-270 10.4056/sigs.3316719 23408774 PMC3569388

[23] Lagier JC Karkouri K Rivet R Couderc C Raoult D Fournier PE Non contiguous-finished genome sequence and description of *Senegalemassilia anaerobia* gen. nov., sp. nov. Stand Genomic Sci 2013; 7:343-356 10.4056/sigs.3246665 24019984 PMC3764928

[24] Mishra AK Hugon P Nguyen TT Robert C Couderc C Raoult D Fournier PE Non contiguous-finished genome sequence and description of *Peptoniphilus obesi* sp. nov. Stand Genomic Sci 2013; 7:357-369 10.4056/sigs.32766871 24019985 PMC3764929

[25] Mishra AK Lagier JC Nguyen TT Raoult D Fournier PE Non contiguous-finished genome sequence and description of *Peptoniphilus senegalensis* sp. nov. Stand Genomic Sci 2013; 7:370-381 10.4056/sigs.3366764 24019986 PMC3764932

[26] Lagier JC Karkouri K Mishra AK Robert C Raoult D Fournier PE Non contiguous-finished genome sequence and description of *Enterobacter massiliensis* sp. nov. Stand Genomic Sci 2013; 7:399-412 10.4056/sigs.3396830 24019988 PMC3764934

[27] Hugon P Ramasamy D Rivet R Raoult D Fournier PE Non contiguous-finished genome sequence and description of *Alistipes obesi* sp. nov. Stand Genomic Sci 2013; 7:427-439 10.4056/sigs.3336746 24019990 PMC3764931

[28] Hugon P Mishra AK Nguyen TT Raoult D Fournier PE Non-contiguous finished genome sequence and description of *Brevibacillus massiliensis* sp. nov. Stand Genomic Sci 2013; 8:1-14 10.4056/sigs.3466975 23961307 PMC3739172

[29] Mishra AK Hugon P Nguyen TT Raoult D Fournier PE Non contiguous-finished genome sequence and description of *Enorma massiliensis* gen. nov., sp. nov., a new member of the Family Coriobacteriaceae. Stand Genomic Sci 2013; 8:290-305 10.4056/sigs.3426906 23991260 PMC3746427

[30] Ramasamy D Lagier JC Gorlas A Raoult D Fournier PE Non contiguous-finished genome sequence and description of *Bacillus massiliosenegalensis* sp. nov. Stand Genomic Sci 2013; 8:264-278 10.4056/sigs.3496989 23991258 PMC3746431

[31] Ramasamy D Lagier JC Nguyen TT Raoult D Fournier PE Non contiguous-finished genome sequence and description of *Dielma fastidiosa* gen. nov., sp. nov., a new member of the Family Erysipelotrichaceae. Stand Genomic Sci 2013; 8:336-351 10.4056/sigs.3567059 23991263 PMC3746426

[32] Mishra AK Pfleiderer A Lagier JC Robert C Raoult D Fournier PE Non contiguous-finished genome sequence and description of *Bacillus massilioanorexicus* sp. nov. Stand Genomic Sci 2013; 8:465-479 10.4056/sigs.4087826 24501631 PMC3910694

[33] Hugon P Ramasamy D Robert C Couderc C Raoult D Fournier PE Non-contiguous finished genome sequence and description of *Kallipyga massiliensis* gen. nov., sp. nov., a new member of the family *Clostridiales Incertae Sedis XI.* Stand Genomic Sci 2013; 8:500-515 10.4056/sigs.4047997 24501634 PMC3910704

[34] Padhmanabhan R Lagier JC Dangui NPM Michelle C Couderc C Raoult D Fournier PE Non-contiguous finished genome sequence and description of *Megasphaera massiliensis.* Stand Genomic Sci 2013; 8:525-538 10.4056/sigs.4077819 24501636 PMC3910696

[35] Stackebrandt E Rainey FA Ward-Rainey NL Proposal for a new hierarchic classification system, *Actinobacteria* classis nov. Int J Syst Bacteriol 1997; 47:479-491 10.1099/00207713-47-2-479

[36] List of Prokaryotic names with standing nomenclature (LPSN). http://www.bacterio.cict.fr

[37] Maruo T Sakamoto M Ito C Toda T Benno Y *Adlercreutzia equolifaciens* gen. nov., sp. nov., an equol-producing bacterium isolated from human faeces, and emended description of the genus *Eggerthella.* Int J Syst Evol Microbiol 2008; 58:1221-1227 10.1099/ijs.0.65404-0 18450717

[38] Minamida K Ota K Nishimukai M Tanaka M Abe A Sone T Tomita F Hara H Asano K *Asaccharobacter celatus* gen. nov., sp. nov., isolated from rat caecum. Int J Syst Evol Microbiol 2008; 58:1238-1240 10.1099/ijs.0.64894-0 18450720

[39] Collins MD Wallbanks S Comparative sequence analyses of the 16S rRNA genes of *Lactobacillus minutus, Lactobacillus rimae and Streptococcus parvulus*: proposal for the creation of a new genus *Atopobium.* FEMS Microbiol Lett 1992; 74:235-240 10.1111/j.1574-6968.1992.tb05372.x 1382033

[40] Kageyama A Benno Y Nakase K Phylogenetic and phenotypic evidence for the transfer of Eubacterium aerofaciens to the genus *Collinsella* as *Collinsella aerofaciens* gen. nov., comb. nov. Int J Syst Bacteriol 1999; 49:557-565 10.1099/00207713-49-2-557 10319476

[41] Haas F König H *Coriobacterium glomerans* gen. nov., sp. nov. from the intestinal tract of the red soldier bug. Int J Syst Bacteriol 1988; 38:382-384 10.1099/00207713-38-4-382

[42] Nakazawa F Poco SE Ikeda T Sato M Kalfas S Sundqvist G Hoshino E *Cryptobacterium curtum* gen. nov., sp. nov., a new genus of gram-positive anaerobic rod isolated from human oral cavities. Int J Syst Bacteriol 1999; 49:1193-1200 10.1099/00207713-49-3-1193 10425779

[43] Anderson RC Rasmussen MA Jensen NS Allison MJ *Denitrobacterium detoxificans* gen. nov., sp. nov., a ruminal bacterium that respires on nitrocompounds. Int J Syst Evol Microbiol 2000; 50:633-638 10.1099/00207713-50-2-633 10758869

[44] Wade WG Downes J Dymock D Hiom S Weightman AJ Dewhirst FE Paster BJ Tzellas N Coleman B The family *Coriobacteriaceae*: reclassification of *Eubacterium exiguum* (Poco et al. 1996) and *Peptostreptococcus heliotrinreducens* (Lanigan 1976) as *Slackia exigua* gen. nov., comb. nov. and *Slackia heliotrinireducens* gen. nov., comb. nov., and *Eubacterium lentum* (Prevot 1938) as *Eggerthella lenta* gen. nov., comb. nov. Int J Syst Bacteriol 1999; 49:595-600 10.1099/00207713-49-2-595 10319481

[45] Clavel T Charrier C Braune A Wenning M Blaut M Haller D Isolation of bacteria from the ileal mucosa of TNFdeltaARE mice and description of *Enterorhabdus mucosicola* gen. nov., sp. nov. Int J Syst Evol Microbiol 2009; 59:1805-1812 10.1099/ijs.0.003087-0 19542111

[46] Würdemann D Tindall BJ Pukall R Lunsdorf H Strompl C Namuth T Nahrstedt H Wos-Oxley M Ott S Schreiber S Timmis KN Oxley AP *Gordonibacter pamelaeae* gen. nov., sp. nov., a new member of the *Coriobacteriaceae* isolated from a patient with Crohn's disease, and reclassification of *Eggerthella hongkongensis* Lau et al. 2006 as *Paraeggerthella hongkongensis* gen. nov., comb. nov. Int J Syst Evol Microbiol 2009; 59:1405-1415 10.1099/ijs.0.005900-0 19502325

[47] Dewhirst FEPaster BJ Tzellas NColeman BDownes JSpratt DAWade WG. Characterization of novel human oral isolates and cloned 16S rDNA sequences that fall in the family *Coriobacteriaceae*: description of *Olsenella* gen. nov., reclassification of *Lactobacillus* *uli* as *Olsenella* *uli* comb. nov. and description of *Olsenella* *profusa* sp. nov. Int J Syst Evol Microbiol 2001; 51:1797-1804 10.1099/ijs.0.005900-0 11594611

[48] Field D Garrity G Gray T Morrison N Selengut J Sterk P Tatusova T Thomson N Allen MJ Angiuoli SV The minimum information about a genome sequence (MIGS) specification. Nat Biotechnol 2008; 26:541-547 10.1038/nbt1360 18464787 PMC2409278

[49] Woese CR Kandler O Wheelis ML Towards a natural system of organisms: proposal for the domains Archae, Bacteria, and Eukarya. Proc Natl Acad Sci USA 1990; 87:4576-4579 10.1073/pnas.87.12.4576 2112744 PMC54159

[50] Garrity GM, Holt JG. The Road Map to the Manual. In: Garrity GM, Boone DR, Castenholz RW (eds), Bergey's Manual of Systematic Bacteriology, Second Edition, Volume 1, Springer, New York, 2001, p. 119-169.

[51] Garrity GM, Holt JG. Taxonomic outline of the *Archae* and *Bacteria* In: Garrity GM, Boone DR, Castenholz RW (eds), Bergey's Manual of Systematic Bacteriology, second edition, Volume, Springer-Verlag, New York, 2001, p. 155-166.

[52] Gupta RS Chen WJ Adeolu M Chai Y Molecular signatures for the class *Coriobacteriia* and its different clades; proposal for division of the class *Coriobacteriia* into the emended order *Coriobacteriales*, containing the emended family *Coriobacteriaceae* and *Atopobiaceae* fam. nov., and *Eggerthellales* ord. nov., containing the family *Eggerthellaceae* fam. nov. Int J Syst Evol Microbiol 2013; 63:3379-3397 10.1099/ijs.0.048371-0 23524353

[53] Zhi XY Li WJ Stackebrandt E An update of the structure and 16S rRNA gene sequence-based definition of higher ranks of the class *Actinobacteria*, with the proposal of two new suborders and four new families and emended descriptions of the existing higher taxa. Int J Syst Evol Microbiol 2009; 59:589-608 10.1099/ijs.0.65780-0 19244447

[54] Ashburner M Ball CA Blake JA Botstein D Butler H Cherry JM Davis AP Dolinski K Dwight SS Eppig JT Gene ontology: tool for the unification of biology. The Gene Ontology Consortium. Nat Genet 2000; 25:25-29 10.1038/75556 10802651 PMC3037419

[55] Seng P Drancourt M Gouriet F La SB Fournier PE Rolain JM Raoult D Ongoing revolution in bacteriology: routine identification of bacteria by matrix-assisted laser desorption ionization time-of-flight mass spectrometry. Clin Infect Dis 2009; 49:543-551 10.1086/600885 19583519

[56] Prodigal. http://prodigal.ornl.gov/

[57] Benson DA Karsch-Mizrachi I Clark K Lipman DJ Ostell J Sayers EW GenBank. Nucleic Acids Res 2012; 40:D48-D53 10.1093/nar/gkr1202 22144687 PMC3245039

[58] Lowe TM Eddy SR tRNAscan-SE: a program for improved detection of transfer RNA genes in genomic sequence. Nucleic Acids Res 1997; 25:955-964 9023104 10.1093/nar/25.5.955 PMC146525

[59] Lagesen K Hallin P Rodland EA Staerfeldt HH Rognes T Ussery DW RNAmmer: consistent and rapid annotation of ribosomal RNA genes. Nucleic Acids Res 2007; 35:3100-3108 10.1093/nar/gkm160 17452365 PMC1888812

[60] Bendtsen JD Nielsen H von Heijne G Brunak S Improved prediction of signal peptides: SignalP 3.0. J Mol Biol 2004; 340:783-795 10.1016/j.jmb.2004.05.028 15223320

[61] Krogh A Larsson B von Heijne G Sonnhammer EL Predicting transmembrane protein topology with a hidden Markov model: application to complete genomes. J Mol Biol 2001; 305:567-580 10.1006/jmbi.2000.4315 11152613

[62] Rutherford K Parkhill J Crook J Horsnell T Rice P Rajandream MA Barrell B Artemis: sequence visualization and annotation. Bioinformatics 2000; 16:944-945 10.1093/bioinformatics/16.10.944 11120685

[63] Carver T Thomson N Bleasby A Berriman M Parkhill J DNAPlotter: circular and linear interactive genome visualization. Bioinformatics 2009; 25:119-120 10.1093/bioinformatics/btn578 18990721 PMC2612626

[64] Darling AC Mau B Blattner FR Perna NT Mauve: multiple alignment of conserved genomic sequence with rearrangements. Genome Res 2004; 14:1394-1403 10.1101/gr.2289704 15231754 PMC442156

[65] Lechner M Findeib S Steiner L Marz M Stadler PF Prohaska SJ Proteinortho: Detection of (Co-)orthologs in large-scale analysis. BMC Bioinformatics 2011; 12:124 10.1186/1471-2105-12-124 21526987 PMC3114741

